# Genomic insight of sulfate reducing bacterial genus *Desulfofaba* reveals their metabolic versatility in biogeochemical cycling

**DOI:** 10.1186/s12864-023-09297-2

**Published:** 2023-04-19

**Authors:** Ping Gao, Xiaoting Zhang, Xiaomei Huang, Zhiyi Chen, Angeliki Marietou, Lars Holmkvist, Lingyun Qu, Kai Finster, Xianzhe Gong

**Affiliations:** 1 Key Laboratory of Marine Eco-Environmental Science and Technology, First Institute of Oceanography, Ministry of Natural Resources (MNR), 266061 Qingdao, PR China; 2 Laboratory for Marine Fisheries Science and Food Production Processes, Pilot National Laboratory for Marine Science and Technology, 266237 Qingdao, PR China; 3 Institute of Marine Science and Technology, Shandong University, 266237 Qingdao, PR China; 4 Section for Microbiology, Department of Biology, Aarhus University, 8000 Aarhus, Denmark; 5 Department of Biological and Chemical Engineering, Aarhus University, 8000 Aarhus, Denmark; 6 Stellar Astrophysics Center, Department of Physics and Astronomy, Aarhus University, 8000 Aarhus, Denmark

**Keywords:** Incomplete sulfate reducer, Desulfofaba, Genome, Oxygen, Sulfur, Nitrogen

## Abstract

**Background:**

Sulfate-reducing bacteria (SRB) drive the ocean sulfur and carbon cycling. They constitute a diverse phylogenetic and physiological group and are widely distributed in anoxic marine environments. From a physiological viewpoint, SRB’s can be categorized as complete or incomplete oxidizers, meaning that they either oxidize their carbon substrate completely to CO_2_ or to a stoichiometric mix of CO_2_ and acetate. Members of *Desulfofabaceae* family are incomplete oxidizers, and within that family, *Desulfofaba* is the only genus with three isolates that are classified into three species. Previous physiological experiments revealed their capability of respiring oxygen.

**Results:**

Here, we sequenced the genomes of three isolates in *Desulfofaba* genus and reported on a genomic comparison of the three species to reveal their metabolic potentials. Based on their genomic contents, they all could oxidize propionate to acetate and CO_2_. We confirmed their phylogenetic position as incomplete oxidizers based on dissimilatory sulfate reductase (DsrAB) phylogeny. We found the complete pathway for dissimilatory sulfate reduction, but also different key genes for nitrogen cycling, including nitrogen fixation, assimilatory nitrate/nitrite reduction, and hydroxylamine reduction to nitrous oxide. Their genomes also contain genes that allow them to cope with oxygen and oxidative stress. They have genes that encode for diverse central metabolisms for utilizing different substrates with the potential for more strains to be isolated in the future, yet their distribution is limited.

**Conclusions:**

Results based on marker gene search and curated metagenome assembled genomes search suggest a limited environmental distribution of this genus. Our results reveal a large metabolic versatility within the *Desulfofaba* genus which establishes their importance in biogeochemical cycling of carbon in their respective habitats, as well as in the support of the entire microbial community through releasing easily degraded organic matters.

**Supplementary Information:**

The online version contains supplementary material available at 10.1186/s12864-023-09297-2.

## Background

Sulfate-reducing bacteria (SRB) are widely distributed in different environments, e.g., wastewater treatment systems [1], freshwater sediments [2], and marine sediments [3]. Dissimilatory sulfate reduction to sulfide by SRB is a predominant terminal pathway of organic matter mineralization in marine sediments [4]. SRB are considered strictly anaerobic, however, it is now generally accepted that a large number of strains tolerate the exposure to oxygen for periods of some length [5–7]. Some members of the genus *Desulfovibrio*, even have high rates of aerobic respiration [6, 8]. Some of them can couple this respiratory process to ATP synthesis [9–11]. Recently, it has been shown that different *Desulfovibrio* strains were able to couple respiration with oxygen to growth [12, 13]. In addition, filamentous sulfide-oxidizing bacteria, the so-called “cable-bacteria”, are able to reduce oxygen (or nitrate) at one end of the filament and oxidize sulfide at the opposing end, thereby transporting electrons over cm-scale distances [14–16]. They represent the only example of a member of the *Desulfobulbaceae* that successfully couples the reduction of oxygen with growth, most likely by reverting the canonical sulfate reduction pathway for the oxidation of sulfide.

SRBs constitute over 23 genera, which are found both within archaeal and bacterial domains with *Deltaproteobacteria* being the dominant class [17]. Recently, some newly discovered bacterial phyla recovered from metagenomic data were found to be SRB [18], suggesting a high phylogenetic diversity of SRB in the environment. However, a large-scale comparison of *Deltaproteobacteria* genomes revealed that the genomes of cultivated *Deltaproteobacteria* strains are phylogenetically distant with those *Deltaproteobacteria* metagenome-assembled genomes (MAGs) [19]. It indicates that the genomic contents of the cultured isolates need further investigation to understand their physiological traits, yet many MAGs are recovered from metagenomic data. By now, only three species, *Desulfofaba fastidiosa* P2 (DSM 15249) [20], *Desulfofaba gelida* PSv29 (DSM 12344) [21], and *Desulfofaba hansenii* P1 (DSM 13527) [22] have been isolated and described within *Desulfofaba*, the genus within *Desulfofabaceae* family. The genus *Desulfofaba* is phylogenetically distinct to other groups of SRBs based on a single gene marker (16S rRNA or DrsA/B). All isolates incompletely oxidizing propionate to acetate and CO_2_ in the presence of sulfate. Interestingly, phylogenetic trees both based on 16S rRNA gene sequences and on dissimilatory sulfite reductase gene amino acid sequences show that the members of the genus *Desulfofaba* are closer related to complete oxidizers than to incomplete oxidizers [20–22].

However, single-gene based study or specific experimental studies may overlook their metabolic potential without exploring their genomic contents. To get a better overview of the metabolic potential of the genus *Desulfofaba*, here we present results of the analysis of the genomes of the three species. We focused on the genes that relate to the handling of oxygen and its intermediates, as physiological experiments with *Desulfofaba hansenii* revealed that the strain was able to respire with oxygen. We also identified genes encoding for the synthesis of polyhydroxybutyrate (PHB) in *Desulfofaba hansenii* and *Desulfofaba gelida*, which potentially could be used as electron donor when oxygen is expired in *Desulfofaba hansenii*. We further investigated the distribution of *Desulfofaba* based on the homologue search of the marker gene, as well as the collection of our MAGs recovered from various marine environments. This is of particular interest as it may provide some clues regarding the habitat adaptation of *Desulfofaba* genus to understand their distribution in the environment.

## Results and discussion

### Phylogeny and distribution of Desulfofaba genus

**Fig. 1:**
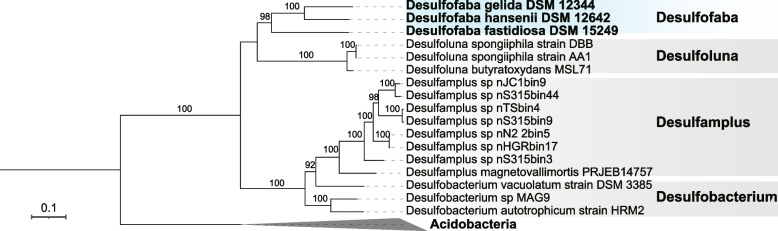
A maximum likelihood phylogenetic tree of 22 genomes including the 3 *Desulfofaba* genomes. The phylogeny is based on 37 concatenated ribosomal protein encoding genes identified using PhyloSift. *Desulfofaba* genomes formed a monophyletic group which is distinct from other families in the order *Desulfobacterales. Acidobacteria* were set as the outgroup

*Desulfofaba* genus consists of three species: *Desulfofaba fastidiosa* P2 (DSM 15249) [20], *Desulfofaba gelida* PSv29 (DSM 12344) [21], and *Desulfofaba hansenii* P1 (DSM 13527) [22]. A set of 120 marker genes identified using GTDB-tk and another set of 37 ribosomal protein encoding marker genes identified using Phylosift supported that *Desulfofaba* is a monophyletic group which is distinct from other families in the order *Desulfobacterales* (Figs. 1 and S1). A phylogenetic tree based on 16S rRNA gene sequences shows that the genus *Desulfofaba* is most closely related to the genus *Desulfoluna* (Fig. S2). The three species of the genus *Desulfofaba* were isolated from different environments, including the interior of an eelgrass root, polar surface sediments, and the methane-sulfate transition zone 1.5 m below the sediment surface [20]. Related 16S rRNA gene sequences recovered from different geographic locations (Fig. S2) suggests a wide distribution of the genus. However, when we searched 16S rRNA gene sequences against publicly available metagenomes, we could not find samples with high sequence homology (> 95%) to 16S rRNA genes. Additionally, we searched *Desulfofaba* genus from 194 *Desulfobacterales* MAGs on phylogenetic trees which were built with protein encoding marker genes extracted using GTDB-tk or Phylosift. Those 194 MAGs were part of our genome collection of over 6000 MAGs. The metagenomes were recovered from various environments, including coastal sediments in the Bohai Sea, cold seep sediments in the South China Sea, and hydrothermal vent sediment in the Southwest Indian Ocean. The search showed that none of them were closely affiliated with members of genus *Desulfofaba*, suggesting that members of the genus *Desulfofaba* are either rare in the environment or difficult to amplify by our current approach.

Average amino acid identity (AAI) revealed that the genus *Desulfofaba* is distinct from other described taxa. These three genomes shared at least 60.1% AAI with each other, and at most 57.5% AAI with genomes of other taxa (Fig. S3).

### Overview of three Desulfofaba genomes

The overall genomic information of the three draft genomes were summarized in Table 1, and the assignment of genes into COG functional categories demonstrating the general function of three genomes is shown in Table 2. The assembled draft genomes are 99.3, 98.1, and 99.3% complete with less than 2.1% contamination based on CheckM [23] for *Desulfofaba fastidiosa*, *Desulfofaba gelida*, and *Desulfofaba hansenii*, respectively.

**Table 1:** Genome statistics of three *Desulfofaba* genomes according to the annotation from IMG

Attribute	*D.fastidiosa*		*D. gelida*		*D. hansenii*	
	Value	% of Total	Value	% of Total	Value	% of Total
Genome size (bp)	3,730,228	100	7,535,424	100	6,711,283	100
DNA coding (bp)	3,259,220	87.37	6,114,098	81.14	5,463,664	81.41
DNA G + C (bp)	1,906,178	51.1	3,926,610	52.11	3,559,937	53.04
DNA scaffolds	147	100	206	100	214	100
Total genes	3503	100	6271	100	5460	100
Protein coding genes	3401	97.09	6046	96.41	5332	97.66
RNA genes	65	1.86	115	1.83	128	2.34
Genes in internal clusters	612	17.47	1416	22.58	1179	21.59
Genes with function prediction	2516	71.82	4339	69.19	3996	73.19
Genes assigned to COGs	2509	71.62	4302	68.6	3011	55.15
Genes with Pfam domains	2591	73.97	4437	70.75	4058	74.32
Genes with signal peptides	137	3.91	342	5.45	296	5.42
Genes with transmembrane helices	825	23.55	1574	25.1	1427	26.14
CRISPR repeats					2

**Table 2:** Number of genes associated with general COG functional categories in three *Desulfofaba* genomes

Description	*D.fastidiosa*		*D. gelida*		*D. hansenii*	
	Value	%age	Value	%age	Value	%age
Amino acid transport and metabolism	175	6.03	413	7.97	333	9.84
Carbohydrate transport and metabolism	116	4	179	3.46	117	3.46
Cell cycle control, cell division, chromosome partitioning	39	1.34	54	1.04	31	0.92
Cell motility	112	3.86	217	4.19	153	4.52
Cell wall/membrane/envelope biogenesis	186	6.41	282	5.44	226	6.68
Chromatin structure and dynamics	2	0.07	2	0.04	1	0.03
Coenzyme transport and metabolism	162	5.58	228	4.4	178	5.26
Cytoskeleton	1	0.03	2	0.04	NA	NA
Defense mechanisms	67	2.31	100	1.93	86	2.54
Energy production and conversion	212	7.3	419	8.09	264	7.8
Extracellular structures	43	1.48	47	0.91	41	1.21
Function unknown	142	4.89	213	4.11	129	3.81
General function prediction only	257	8.85	580	11.2	284	8.39
Inorganic ion transport and metabolism	117	4.03	263	5.08	218	6.44
Intracellular trafficking, secretion, and vesicular transport	68	2.34	86	1.66	58	1.71
Lipid transport and metabolism	92	3.17	177	3.42	130	3.84
Mobilome: prophages, transposons	63	2.17	96	1.85	23	0.68
Nucleotide transport and metabolism	79	2.72	93	1.8	75	2.22
Posttranslational modification, protein turnover, chaperones	151	5.2	211	4.07	142	4.2
RNA processing and modification	1	0.03	NA	NA	NA	NA
Replication, recombination and repair	127	4.37	140	2.7	97	2.87
Secondary metabolites biosynthesis, transport and catabolism	43	1.48	123	2.37	81	2.39
Signal transduction mechanisms	271	9.34	662	12.78	339	10.02
Transcription	144	4.96	333	6.43	157	4.64
Translation, ribosomal structure and biogenesis	233	8.03	260	5.02	221	6.53
Not in COG	994	28.38	1969	31.4	2449	44.85

### Complex organic matter degradation

**Fig. 2:**
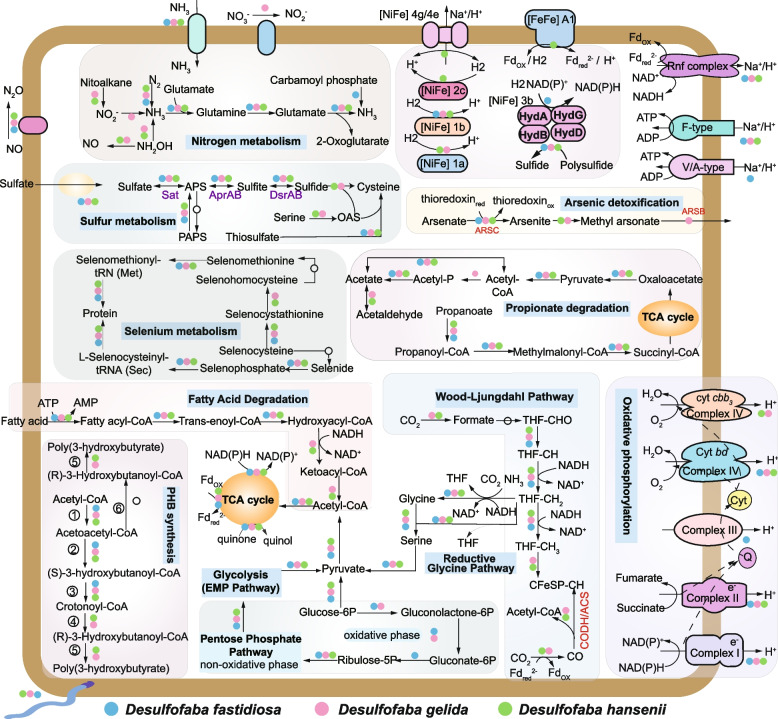
Overview of the metabolic potential of the *Desulfofaba* genus based on the annotated genomes. *Desulfofaba* genomes have genes encoding for diverse central metabolic pathways, including glycolysis, the pentose phosphate pathway (PPP), the Wood-Ljungdahl pathway (WLP), the TCA cycle, and the reductive glycine pathway. *Desulfofaba* genomes have genes involved in nitrogen, sulfur, hydrogen, selenium, and arsenic cycling. Different types of cytochrome oxidases genes were annotated in *Desulfofaba* genomes

These genomes include genes encoding for diverse carbohydrate-active enzymes (CAZYmes) and peptidases (Fig. S4) with the potential for degradation of complex carbohydrates and detrital proteins into simple sugars and amino acids. Most of the enzymes are assigned with intra-cellular function based on predictions of the cellular localization of the proteins. They also have genes encoding for proteins that are capable of degrading long-chain fatty acids through beta oxidation (Fig. 2), which is consistent with experimental data for *Desulfofaba gelida* [21]. As an incomplete oxidizer [20–22], they are capable of oxidizing propionate to acetate with the methylmalonyl-CoA pathway, which shares several steps with the tricarboxylic acid (TCA) cycle (Fig. 2). The acetate is excreted and may serve as a substrate for adjacent microbes in the environment.

### Central metabolism and PHB synthesis

Genes encoding central metabolic pathways, including glycolysis, the pentose phosphate pathway (PPP), the Wood-Ljungdahl pathway (WLP), the TCA cycle, and the reductive glycine pathway were identified (Fig. 2, Supplementary Dataset). This suggests a high degree of metabolic versatility of central metabolism in *Desulfofaba* genus, which enables them to utilize different types of substrates or be active under different environmental conditions.

All three *Desulfofaba* genomes have a pathway for PHB synthesis from acetyl-CoA [24, 25]. Genes encoding for acetoacetyl-CoA reductase (PhaB), reducing acetoacetyl-CoA to 3-hydroxybutyryl-CoA, were not annotated in any of these three genomes. However, genes encoding for 3-hydroxybutyryl-CoA dehydrogenase, enoyl-CoA hydratase, and 3-hydroxybutyryl-CoA dehydratase, which could reduce acetoacetyl-CoA through 3-hydroxybutanoyl-CoA and crotonoyl-CoA, were annotated in *Desulfofaba* genomes (Fig. 2).

### Sulfur and nitrogen metabolism

**Fig. 3:**
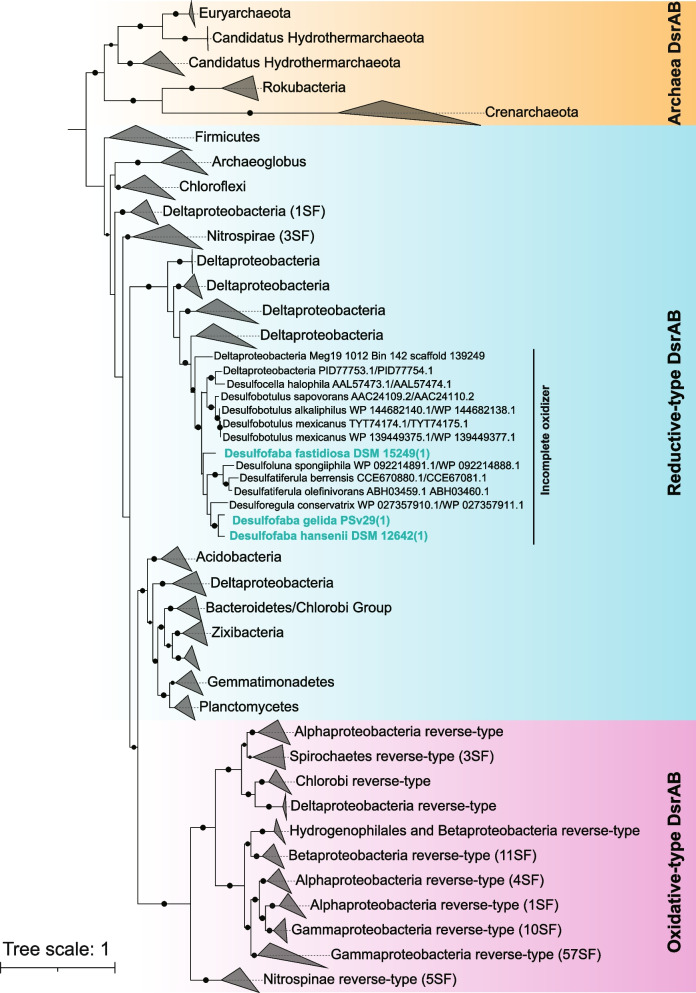
A maximum likelihood phylogenetic tree of genes encoding for alpha and beta subunits of dissimilatory sulfite reductase (DsrAB). DsrAB in *Desulfofaba* genomes belong to reductive-type and are closely related to sequences in known incomplete oxidizer genomes

The three species within *Desulfofaba* genus are incomplete oxidizers, i.e., oxidizing propionate incompletely to acetate and CO_2_ [20–22]. The phylogenetic tree of dissimilatory sulfite reductase (DsrAB) genes showed that the three species, together with other identified incomplete oxidizers, formed a monophyletic group (Fig. 3). This phylogenetic position contradicts with the previous study, which showed a closer phylogeny with complete oxidizers based on 16S rRNA gene or DSR sequencing [22]. According to their genetic inventory, they have the potential to reduce polysulfide to sulfide using a sulfhydrogenase and to assimilate thiosulfate into cysteine.

The three genomes also encode several key pathways for nitrogen cycling (Fig. 2) such as nitrogenase genes involved in the fixation of nitrogen. Also, genes encoding the assimilation of ammonia into glutamine were found. On the dissimilatory side, *Desulfofaba gelida* encodes genes for nitrate/nitrite assimilation. Hydroxylamine is an intermediate in two important microbial processes of the nitrogen cycle: nitrification [26] and anaerobic ammonium oxidation [27]. The genomes of both *Desulfofaba gelida* and *Desulfofaba hansenii* encode enzymes to oxidize hydroxylamine (NH_2_OH) to nitric oxide (NO) via hydroxylamine dehydrogenase (HAO) [28, 29] or reduce NH_2_OH to ammonia (NH_3_) via hydroxylamine reductase (HCP). All genomes encode enzymes to further reduce NO to nitrous oxide (N_2_O) via anaerobic nitric oxide reductase. N_2_O is a key byproduct during denitrification and is a potent greenhouse gas and ozone destroying agent. This indicates that *Desulfofaba* may have important implications for Earth’s climate [30].

### Hydrogen metabolism and energy conservation

Hydrogen plays a central role in the energy metabolism of sulfate reducers, which can either use H_2_ as an energy source or produce H_2_ during fermentation [31]. The genomes of three species of the genus *Desulfofaba* encode [NiFe] group 1b hydrogenase, which may transfer H_2_-liberated electrons through cytochromes to terminal reductase when sulfate, fumarate, nitrate, and metals serve as terminal electron acceptors [32]. The presence of other types of [NiFe] and [FeFe] hydrogenase genes, including cytoplasmic and transmembrane types for hydrogen metabolism, is species-specific (Fig. 2). The phylogenetic position of *Desulfofaba* hydrogenases is close to hydrogenases of other known sulfate reducers (Figs. S5 and S6). Besides genes encoding for respiratory complex I and II in the electron transport chain, they also encode the membrane-bound Rnf complex that can couple the electron transfer from reduced ferredoxin (Fd^2−^) to NAD^+^ with the translocation of proton (H^+^) for energy conservation [33]. The three species encode F-type ATPase to generate ATP (Fig. 2).

### Selenium and arsenate metabolism

The genomes of the three *Desulfofaba* species have genes encoding for proteins involved in the mobilization of organic selenium. Those proteins catalyze the reaction of selenide with ATP to form selenophosphate via selenide water dikinase (SelD) [34] and incorporate selenium into selenocysteinyl-tRNA (Sec) with L-seryl-tRNA (Ser) selenium transferase (SelA) [35] and selenomethionyl-tRNA (Met) with methionyl-tRNA synthetase (MetG) [36], which are further utilized for protein biosynthesis.

Arsenate and arsenite are toxic to organisms by blocking general cell metabolism [37] and are the two dominant forms of inorganic arsenic in marine environments [38]. *Desulfofaba* has genes of the arsenic detoxification system (Fig. 2). They can reduce arsenate to arsenite via arsenate reductase (ArsC) through thioredoxin [39]. Even though arsenite is more toxic than arsenate, arsenite could be extruded from the cell by an arsenite transporter (ArsAB) or transformed to methyl arsonate, a less toxic form [40], by arsenite methyltransferase (AS3MT).

### Oxygen Consumption and Defense against ROS

Most sulfate reducers are obligate anaerobes, yet some species can tolerate oxygen and have developed different strategies to cope with its presence in the environment [41]. We identified genes encoding two types of membrane-bound oxygen reductases: cytochrome *bd*-I ubiquinol oxidase and cytochrome *cbb*_*3*_-type terminal oxidase (Fig. 2). The first evidence for a membrane-bound oxygen reductase, a canonical *bd* quinol oxidase, in SRB was reported in *Desulfovibrio gigas* [11]. The genes of both identified terminal oxidases in *Desulfofaba* are of a high-affinity-type, and they are usually considered as important terminal oxidases under low oxygen conditions [42]. Our physiological experiments showed that *Desulfofaba hansenii* was able to reduce oxygen and that oxygen reduction is most likely linked to the oxidation of PHB storage compounds that are present in the cell (Supplementary material). Many sulfate reducers can reduce O_2_, probably as a protective mechanism, without sustainable aerobic growth [43]. Growth with energy derived from oxidative phosphorylation linked to oxygen reduction was observed in different *Desulfovibrio* strains [12, 13]. Based on their genomic outfit, it is possible that *Desulfofaba* sp. produces energy during aerobic respiration. However, we did not observe aerobic respiration linked to growth in *Desulfofaba hansenii*. Both *Desulfofaba hansenii*, isolated from *Zostera marina* roots, and *Desulfofaba gelida*, isolated from surface sediments, could encounter oxygen in their respective habitats and thus, the ability to cope with oxygen could be an important survival strategy.

The genomes that we studied contain a number of genes that enable them to deal with oxidative stress, as is the case for many other sulfate reducers [44]. These include genes to detoxify reactive oxygen species (ROS) and repair damaged DNA, as well as genes that trigger a behavioral response to the presence of O_2_ and ROS. ROS, including superoxide, hydrogen peroxide and hydroxyl radical, are formed during oxygen reduction in the oxygen reduction systems and during non-specific reactions of oxygen with reduced substrates, e.g., transition metals and radical species [45]. The toxicity of O_2_ in cells is mainly due to cellular damages caused by ROS, such as the oxidation of thiols and the release of metallic centers from proteins leading to the increase of free metals in cytosol. The increased free metals, mainly iron, cause DNA damage through fenton-type reactions that produce ROS [44]. The removal of ROS is important for cells to deal with oxidative stress. We found genes encoding nickel-containing superoxide dismutase that eliminates superoxide radicals through disproportionation into H_2_O_2_ and O_2_ [46] and superoxide reductase that converts toxic O_2_^−^ into less toxic H_2_O_2_ [47]. These genes have been found in other SRB, e.g., in *Desulfovibrio* genus [48, 49]. In addition, genes encoding enzymes that can decompose H_2_O_2_ [47], such as catalase, thiol peroxidase, cytochrome *c* peroxidase, peroxiredoxin, and rubrerythrin, were found. Catalase is a common enzyme in aerobic organisms that catalyzes the detoxification of H_2_O_2_ and has been found in many sulfate reducers both the bacterial and archaeal domains e.g., *Desulfovibrio gigas* [50] and *Archaeoglobus fulgidus* [51],

The investigated genomes encode several damage repair systems: (1) thioredoxin, thioredoxin reductase, and glutaredoxin for disulphide bonds reductions; (2) methionine sulfoxide reductase (MsrA/MsrB) for oxidized methionines reduction; and (3) NifU like protein for the Fe-S clusters respiration or biosynthesis (Supplementary Dataset) [44]. The enzymes involved in the damage repair system are metal-free. In contrast, the damage in the detoxification system (superoxide reductase and rubrerythrin) contributes to an increase of Fe^2+^, leading to the formation of ROS, which requires more repair. Therefore, the system for damage repair is more important than the detoxification system in cells when oxidative conditions are more severe. For example, the expression gene that encodes enzymes of damage repair systems were highly upregulated in *Desulfovibrio vulgaris* after oxidative stress [52]. Apart from mechanisms dealing with oxidative stress, studied genomes encode methyl-accepting chemotaxis proteins (MCP), which are involved in behavioral strategies to avoid and thereby protect cells from contact with oxygen [10, 43, 53, 54].

### Comparative genomics

**Fig. 4:**
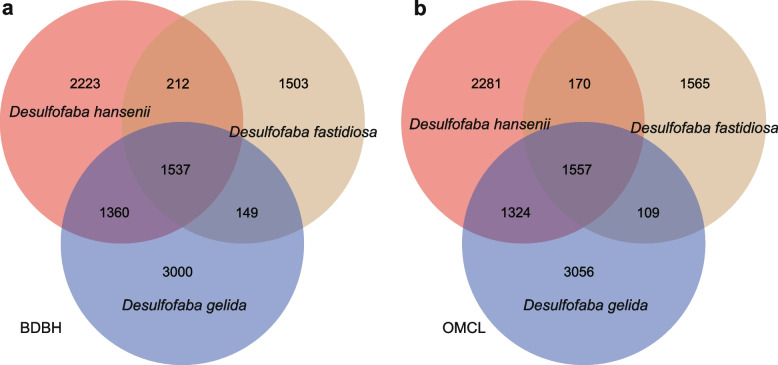
Shared protein contents among three *Desulfofaba* genomes based on two different algorithms: **a** bidirectional best-hits (BDBH) and **b** orthoMCL algorithm (OMCL). Over 1500 proteins were shared between the three species, and over 1/3 of protein sequences in each genome were species-specific

The three studied genomes share 1557 and 1537 homologues which is at least 1/3 of their genome content based on two algorithms with BDBH and OMCL options (Fig. 4). These homologues have key functions for cell maintenance processes. There are still large portions of species-specific proteins, over 1/3 of proteins in each genome that do not have homologues in the other two species, that may be attributed to the different isolation sources of these three species. The number of proteins in *Desulfofaba fastidiosa* shared with the other two species were much lower than number of genes shared between *Desulfofaba hansenii* and *Desulfofaba gelida*. This potential syntrophic process of anaerobic oxidation of methane coupled to sulfate reduction in the sulfate-methane transition zone may increase the chance for lateral gene transfer, which further contributes to the smaller genome size in *Desulfofaba fastidiosa* [55]. Genes related with transcription, signal transduction, and secondary metabolite synthesis tend to lose during genome reduction in symbiotic genomes [56]. Moreover, the numbers of transport genes are positively correlated with *Desulfofaba* genome sizes (Table 2), which is consistent with the universal relationship with genome size [56]. It further suggests that the sulfate-methane transition zone in marine sediment, where *Desulfofaba fastidiosa* was isolated from, is more different from the other two environments, e.g., seagrass roots and surface sediment.

## Conclusions

Strains belonging to the *Desulfofaba* genus were isolated from different environments. Genomic content of this genus showed a high degree of versatility of central metabolic pathways. The diverse central metabolism indicates that isolates have the potential to utilize a wide range of substrates. The presence of different genes involved in sulfur and nitrogen metabolism suggest that they may play a role in various aspects of sulfur and nitrogen cycling. A 16S rRNA gene homologues-based search in publicly available metagenomes and our collection of MAGs from various marine environments indicates a limited environmental distribution of this genus. This, however, does not exclude members of *Desulfofaba* genus from having a significant role in biogeochemical cycling in their respective habitats. Their ability to respire oxygen and the presence of genes for ROS damage defense allows them to inhabit environments with regular oxygen intrusion, such as the roots of *Zostera marina* plants from which *Desulfofaba hansenii* was isolated from. The incomplete oxidation of propionate to acetate provides easily utilized electron donors to other microbes which benefits the entire microbial community.

## Methods

### Bacteria cultivation and DNA extraction

*Desulfofaba fastidiosa* P2 (DSM 15249) [20], *Desulfofaba gelida* PSv29 (DSM 12344) [21], and *Desulfofaba hansenii* P1 (DSM 13527) [22] were grown as described in the literature [20–22]. The cells were harvested in the late exponential phase. DNA was extracted from the cell pellet using the PowerLyser® PowerSoil® DNA extraction kit (MoBio, Carlsbad, CA, USA) according to the manufacturer’s protocol.

### Genome sequencing, assembly, annotation, and homologues search

DNA was sequenced on a MiSeq platform. The sequencing data were treated as previously described [57]. Briefly, the sequencing library was trimmed with Trimmomatic-0.36 [58] with the following trimming parameters: CROP:290 HEADCROP:25 SLIDINGWINDOW:4:20. Read quality before and after trimming was assessed by FastQC version 0.11.5 (http://www.bioinformatics.babraham.ac.uk/projects/fastqc/). Reads were assembled using SPAdes 3.10.1 [59]. Contigs shorter than 1,000 bp were removed after assembling. The quality of the assembled genomes was estimated using CheckM v1.1.3 [23]. The draft genome was annotated using the standard operation procedure of the DOE-JGI Microbial Genome Annotation Pipeline (MGAP v.4) supported by the JGI (Berkeley, CA; USA) [60]. The predicted protein sequences from MGAP were further annotated using KofamScan v.1.3.0 with the e-value cut-off of 1e-5 and the highest bit-score larger than the pre-set threshold for each gene [61], and further characterized using KAAS (KEGG Automatic Annotation Server) web server [62] using the ‘Complete or Draft Genome’ setting with parameters: GHOSTX, custom genome dataset, and BBH assignment method.

Key metabolic genes were searched using custom databases. Briefly, peptidases were identified using DIAMOND BLASTP v0.9.31.132 [63] to search against MEROPS Peptidase Protein Sequences (Downloaded on 24^th^, March, 2022) [64] with the settings: -e 1e-10 –subject-cover 80 –id 50 [65]. Carbohydrate active enzymes (CAZYmes) were identified using the dbCAN v2 standalone tool (CAZYDB.09242021, dbCAN-HMMdb-V10, and dbCAN-fam-aln-V9 databases) [66] with default thresholds. The localization of identified peptidases and CAZYmes was determined using the command-line version of Psort v3.0 using the option –negative for the genomes.

Genes encoding for dissimilatory sulfite reductase (DsrAB) and hydrogenase were further identified using DIAMOND BLSATP v0.9.31.132 [63] to search against different custom databases with the thresholds: -e 1e-10 –subject-cover 70 –id 50; -e 1e-10 –subject-cover 50 –id 30; and -e 1e-10 –subject-cover 50 –id 40 for DsrAB and hydrogenase sequences, respectively. The identified sequences were confirmed with the annotation from MGAP and KO assignment. The identified hydrogenase sequences were further compared with the annotation based on the assigned KO number and the web-based hydrogenase classifier (26^th^ March, 2022) [32].

Homologues between different genomes were searched by GET_HOMOLOGUES [67, 68] with BDBH and OMCL options.

### Phylogenetic analysis

A set of 120 marker genes was extracted from the three genomes and reference genomes using the Genome Taxonomy Database (GTDB)-Tk v1.7.0 (release 202) [69]. Another set of 37 single-copy, protein-coding housekeeping genes was extracted using Phylosift v1.0.1 [70]. The two sets of marker genes were separately concatenated, and aligned using MAFFT v7.450 [71] with the setting –maxiterate 1000 –localpair, trimmed using trimAl v1.2rev59 [72] with the -gappyout option, and manually checked. The two refined alignments were used to generate two maximum-likelihood trees using RAxML v8.2.4 [73] with the parameters: raxmlHPC-PTHREADS-AVX -m GTRGAMMA -N autoMRE -p 12345—× 12345. Amino acid identity (AAI) of the three genomes and reference genomes was estimated using CompareM (v0.1.2) AAI workflow (‘comparem aai_wf’, https://github.com/dparks1134/CompareM).

16S rRNA sequences were identified using Barrnap v0.9 (https://github.com/tseemann/barrnap) with the default settings, aligned, and manually curated in ARB [74] with the SILVA SSURef NR99 database (release 138). The alignment was exported to generate a maximum-likelihood tree using IQ-TREE v1.6.12 [75] with the settings: -m MFP -bb 1000 -bnni -alrt 1000.

The identified DsrA and DsrB sequences were separately aligned with reference sequences using MAFFT v7.450 with the settings: –maxiterate 1000 –globalpair –anysymbol, trimmed using trimAl v1.2rev59 [72] with the -gappyout option, manually checked, and concatenated. The maximum-likelihood tree was generated using RAxML v8.2.4 [73] with the parameters: raxmlHPC-PTHREADS-AVX -m GTRGAMMA -N autoMRE -p 12345—× 12345.

The final identified hydrogenase sequences with selected references for different types of hydrogenases [76] were aligned using ClustalW v2.1 [77], and the Neighbor-Joining tree was generated using MEGA X [78] under p-distance model with 1,000 bootstrap.

## Supplementary Information

**Additional file 1: Figure S1.** A maximum likelihood phylogenetic tree of xx genomes including the 3 *Desulfofaba* genomes. The phylogeny is based on 120 concatenated ribosomal protein encoding genes identified using GTDB-tk. Acidobacteria were set as the outgroup.

**Additional file 2: Figure S2.** Maximum likelihood phylogenetic tree of 16S rRNA gene.

**Additional file 3: Figure S3.** Hierarchical clustering heatmap using pheatmap package in R based on average amino acids identity (AAI) for each genome pair.

**Additional file 4: Figure S4.** Carbohydrate-active enzymes (CAZyme) and peptidase encoded by *Desulfofaba* genus. (a) CAZymes include carbohydrate esterase (CE), glycoside hydrolase (GH), and polysaccharide lyase (PL). (b) Peptidases are classified by family as aspartic (A), cysteine (C), unassigned inhibitors (I), metallo (M), asparagine (N), serine (S), threonine (T), and unknown (U) by the MEROPS database. Sizes of the circle denote the number of gene copies in the genome. The number on top of the circle represents the number of sequences identified with potential secretion signal using PSORTb v3.0.

**Additional file 5: Figure S5.** Maximum likelihood phylogenetic tree of NiFe hydrogenases. Bootstrap values ≥ 75 are shown in circles.

**Additional file 6: Figure S6.** Maximum likelihood phylogenetic tree of FeFe hydrogenases. Bootstrap values ≥ 75 are shown in circles.

**Additional file 7: Figure S7.** Schematic drawing of the reaction chambers used in this study. Type I chamber (a) was used at oxygen concentrations between 0 and 36 μM; type II chamber (b) was used up to 140 μM.

**Additional file 8: Figure S8.** Oxygen respiration under different conditions. (a) Oxygen consumption rates under different initial oxygen concentrations. The oxygen consumption rates increased with increasing oxygen concentrations up to about 40 μM and decreased slowly to the lowest rates at 140 μM oxygen. Filled circles represent rates obtained in type I chamber, while open circles represent rates obtained in type II chamber. (b) Oxygen consumption started immediately after the oxygenated medium was injected (final concentration 36 μM) into the culture. The highest rates were measured in the beginning of the monitoring period. (c) The effect of formate on the rate of oxygen consumption. The experiment was initiated by injection of oxygenation medium (final concentration 25 μM of oxygen). The immediate consumption rate of oxygen was 15 nmol O_2_ min^-1^ mg protein^-1^. After addition of formate (final concentration 20 mM) the oxygen consumption rate increased to 22 nmol O_2_ min^-1^ mg protein^-1^.

**Additional file 9: Figure S9.** A Lineweaver-Burk plot constructed from the first eleven measurements shown in Fig. S8a. The X and Y intercepts are used to calculate *K*_*m*_ and *V*_*max*_.

**Additional file 10: Figure S10.** Cells of *D*. *hansenii* before (a) and after (b) exposure to 60 μM oxygen for 20 h. The cells were stained with Nile blue, which binds to polyhydroxyalkanoates. The red color represents areas in the cells, which were stained by Nile blue, indicating that the amount of polyhydroxyalcanoates decreased after exposure to oxygen.

**Additional file 11.** Detailed annotation of the three *Desulfofaba* genomes.

**Additional file 12.**

## Data Availability

The genome sequence and annotation can be found in IMG/JGI under the IMG Genome ID: 2928859439, 2740891818, and 2929304470.

## Abbreviations

SRB: Sulfate-reducing bacteria
Dsr: Dissimilatory sulfite reductase
MAGs: Metagenome-assembled genomes
PHB: Polyhydroxybutyrate
AAI: Average amino acid identity
CAZYmes: Carbohydrate-active enzymes
TCA: Tricarboxylic acid
PPP: Pentose phosphate pathway
WLP: Wood-Ljungdahl pathway (WLP)
NH_2_OH: Hydroxylamine
NO: Nitric oxide
HAO: Hydroxylamine dehydrogenase
NH_3_: Ammonia
HCP: Hydroxylamine reductase
N_2_O: Nitrous oxide
Fd^2^^−^: Ferredoxin
H^+^: Proton
ROS: Reactive oxygen species
MCP: Methyl-accepting chemotaxis proteins
MGAP: Microbial Genome Annotation Pipeline
KAAS: KEGG Automatic Annotation Server
GTDB: Genome Taxonomy Database
