# Supplementary figures and images for: Genomic insight of sulfate reducing bacterial genus Desulfofaba reveals their metabolic versatility in biogeochemical cycling
Source: BMC Genomics. 2023 Apr 19;24:209. doi: 10.1186/s12864-023-09297-2 (PMC10116758; doi:10.1186/s12864-023-09297-2)

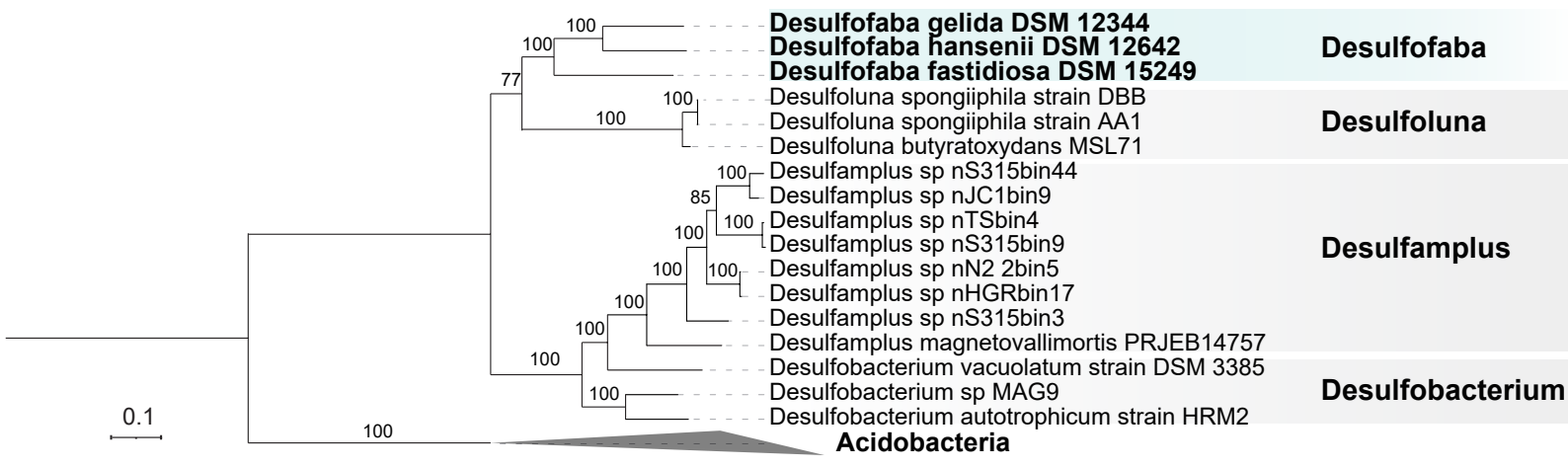

Supplement: Supplementary file 1 — Additional file 1: Figure S1. A maximum likelihood phylogenetic tree of xx genomes including the 3 Desulfofaba genomes. The phylogeny is based on 120 concatenated ribosomal protein encoding genes identified using GTDB-tk. Acidobacteria were set as the outgroup. [file 12864_2023_9297_MOESM1_ESM.pdf]

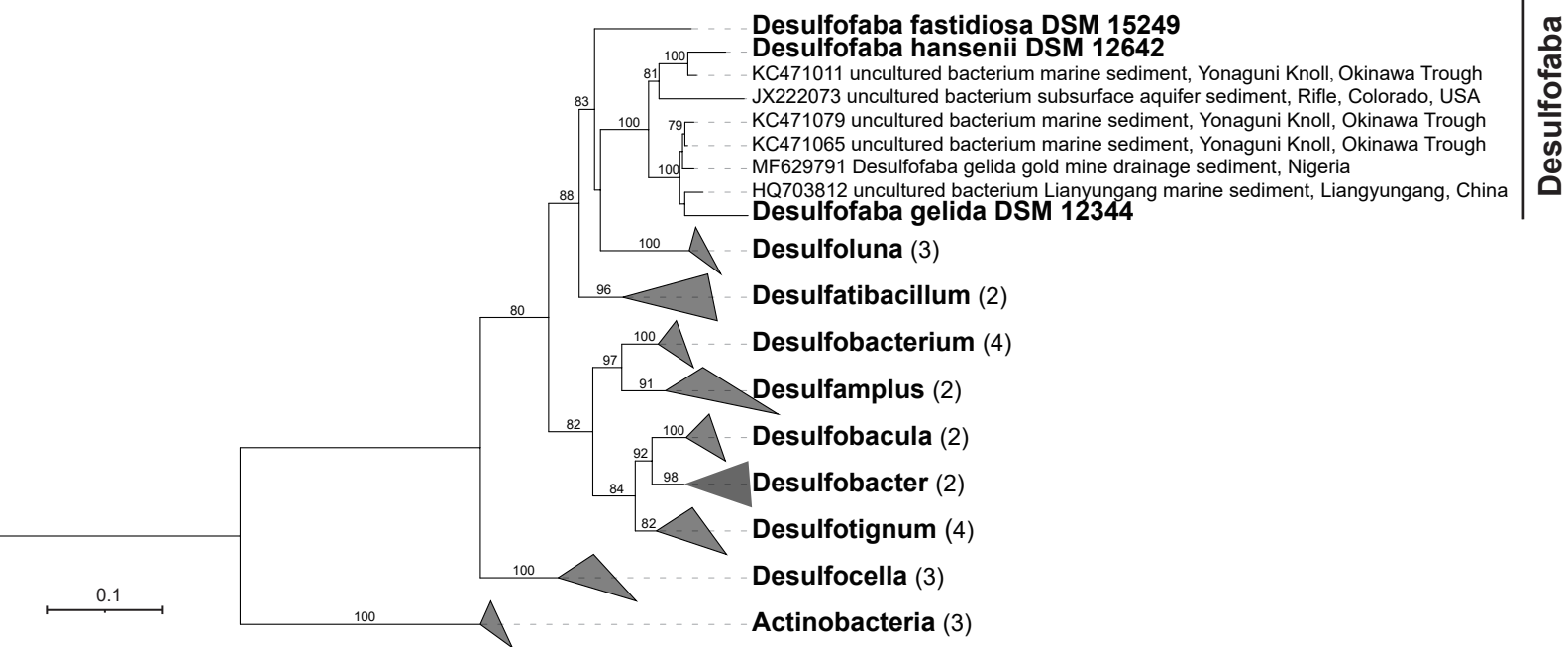

Supplement: Supplementary file 2 — Additional file 2: Figure S2. Maximum likelihood phylogenetic tree of 16S rRNA gene. [file 12864_2023_9297_MOESM2_ESM.pdf]

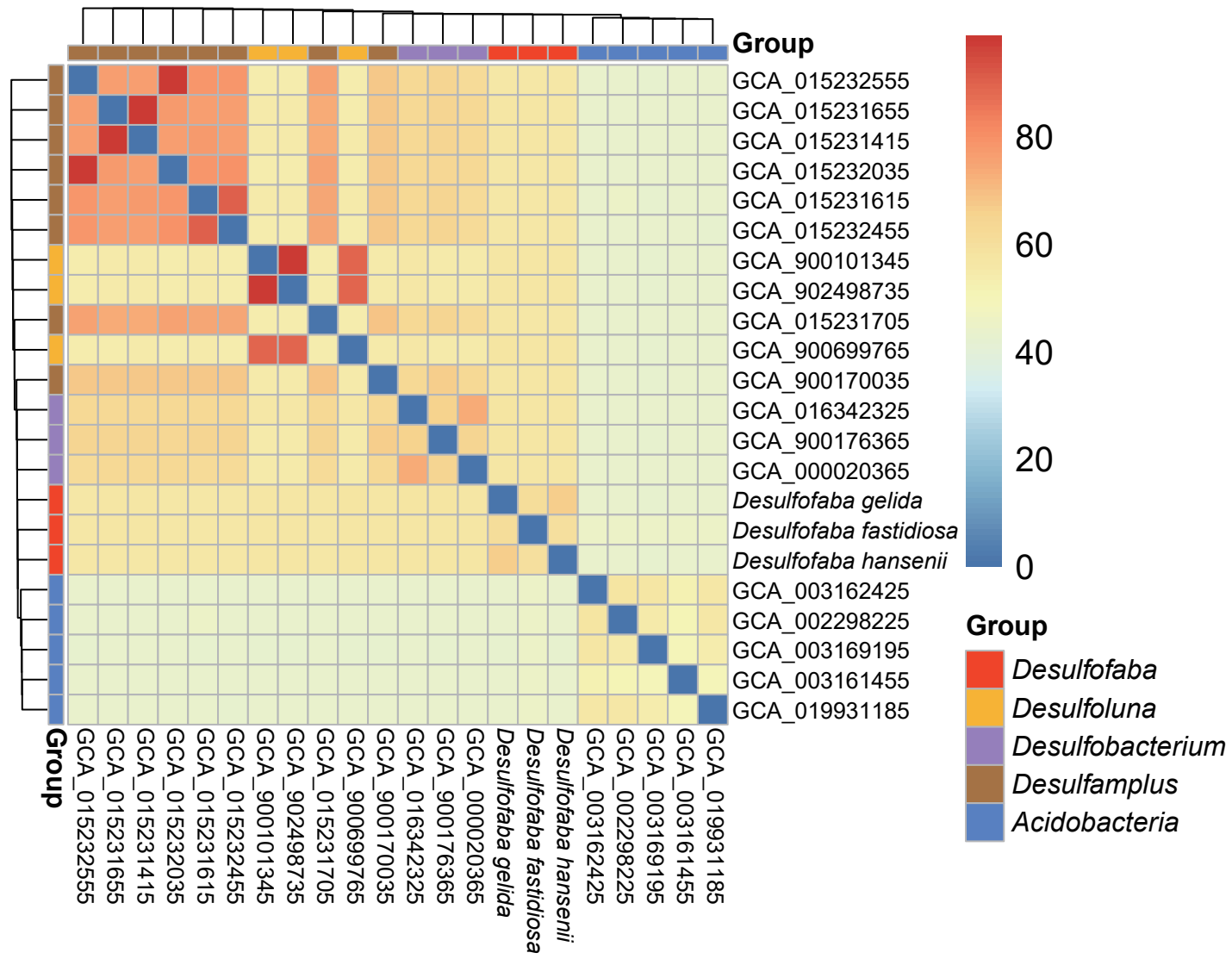

Supplement: Supplementary file 3 — Additional file 3: Figure S3. Hierarchical clustering heatmap using pheatmap package in R based on average amino acids identity (AAI) for each genome pair. [file 12864_2023_9297_MOESM3_ESM.pdf]

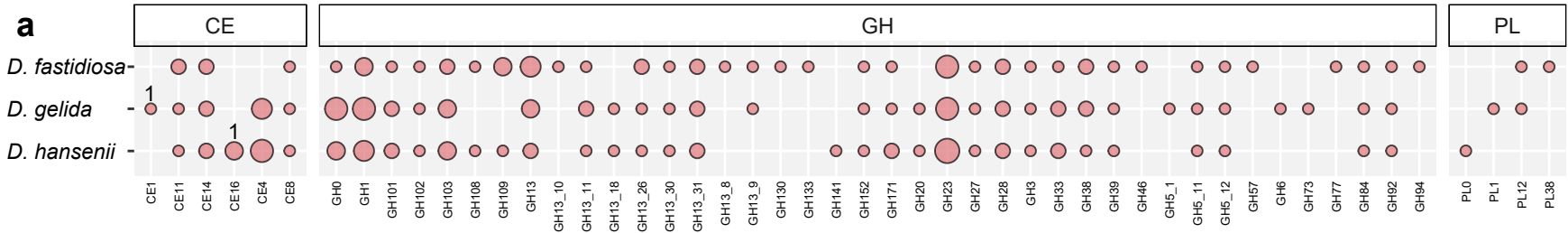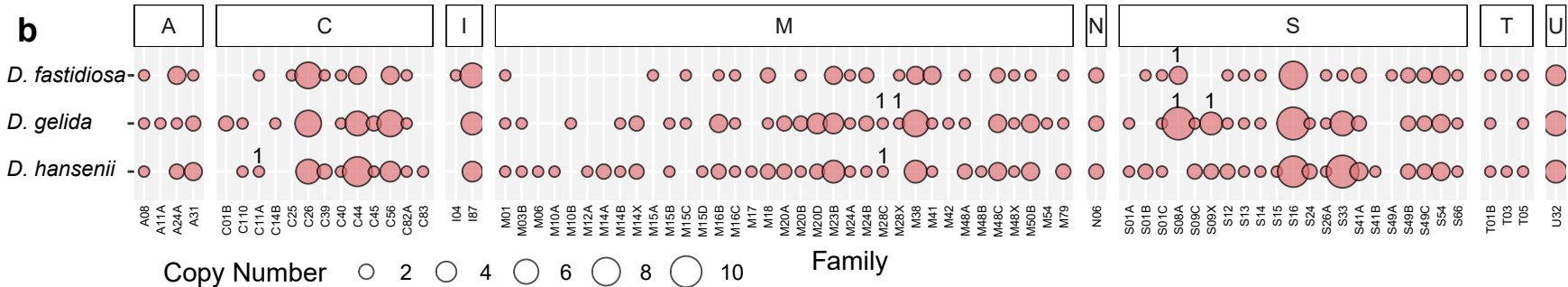

Supplement: Supplementary file 4 — Additional file 4: Figure S4. Carbohydrate-active enzymes (CAZyme) and peptidase encoded by Desulfofaba genus. (a) CAZymes include carbohydrate esterase (CE), glycoside hydrolase (GH), and polysaccharide lyase (PL). (b) Peptidases are classified by family as aspartic (A), cysteine (C), unassigned inhibitors (I), metallo (M), asparagine (N), serine (S), threonine (T), and unknown (U) by the MEROPS database. Sizes of the circle denote the number of gene copies in the genome. The number on top of the circle represents the number of sequences identified with potential secretion signal using PSORTb v3.0. [file 12864_2023_9297_MOESM4_ESM.pdf]

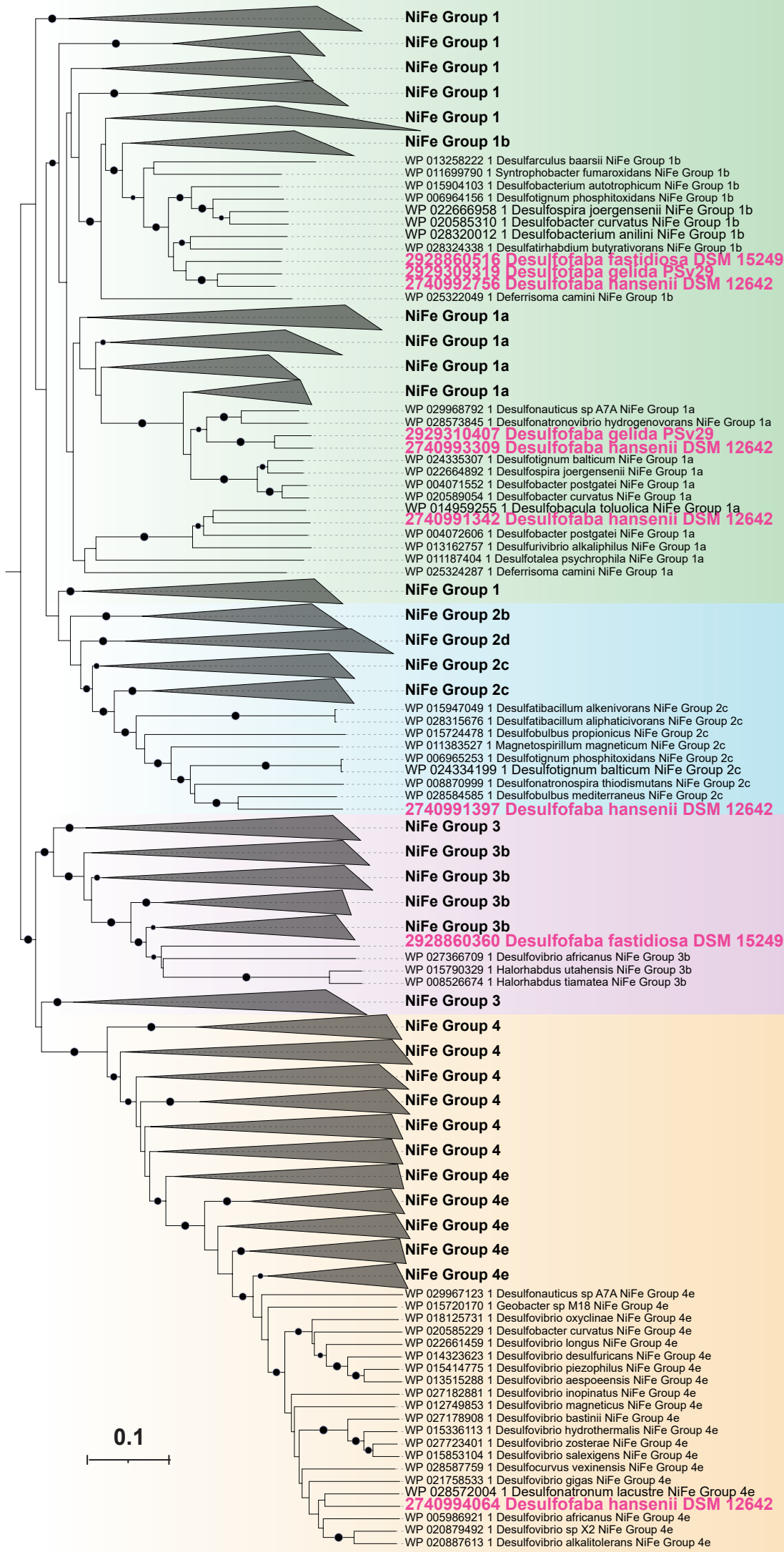

Supplement: Supplementary file 5 — Additional file 5: Figure S5. Maximum likelihood phylogenetic tree of NiFe hydrogenases. Bootstrap values ≥ 75 are shown in circles. [file 12864_2023_9297_MOESM5_ESM.pdf]

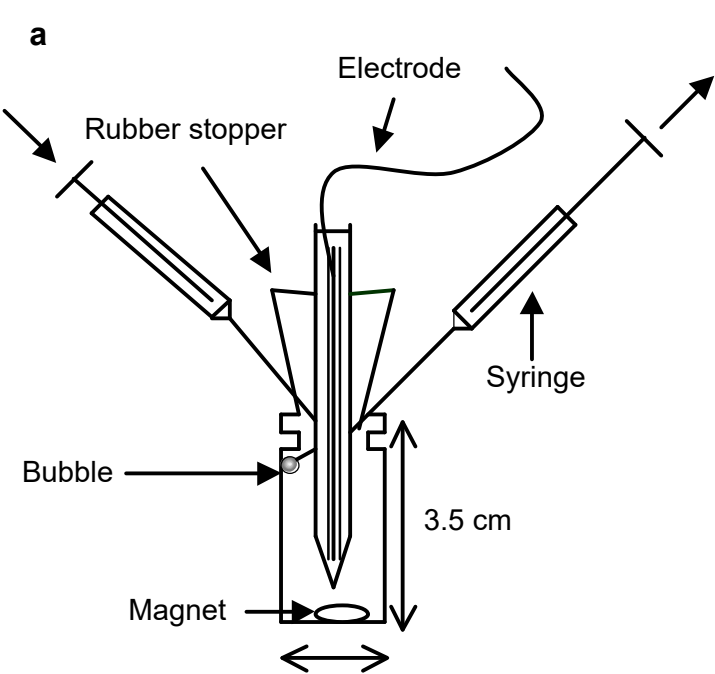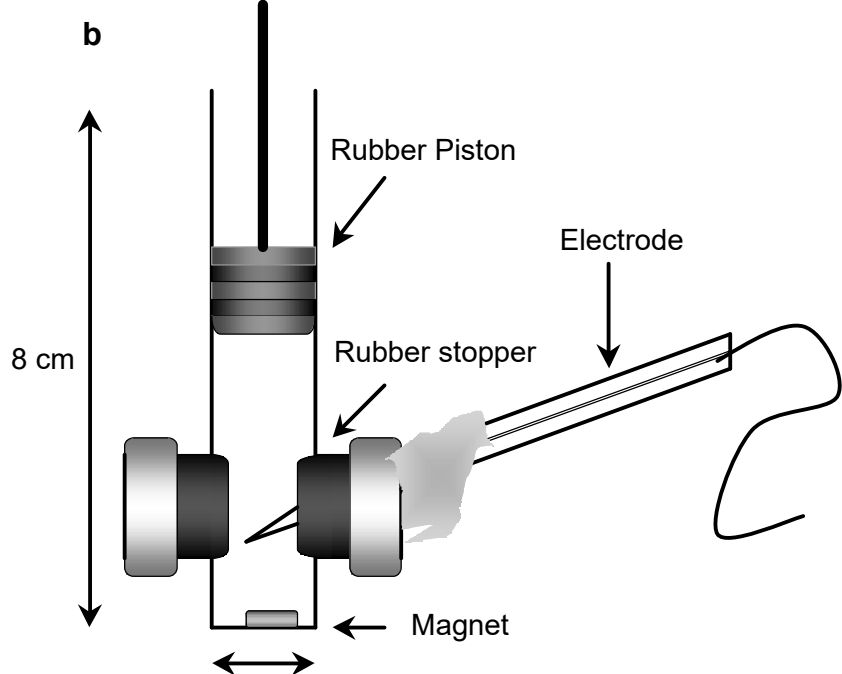

Supplement: Supplementary file 7 — Additional file 7: Figure S7. Schematic drawing of the reaction chambers used in this study. Type I chamber (a) was used at oxygen concentrations between 0 and 36 μM; type II chamber (b) was used up to 140 μM. [file 12864_2023_9297_MOESM7_ESM.pdf]

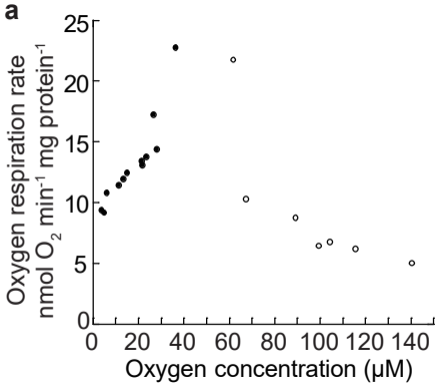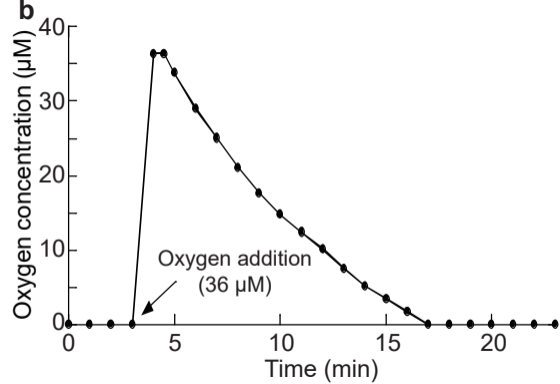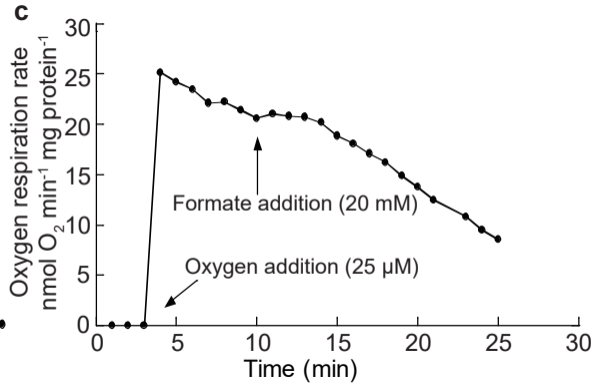

Supplement: Supplementary file 8 — Additional file 8: Figure S8. Oxygen respiration under different conditions. (a) Oxygen consumption rates under different initial oxygen concentrations. The oxygen consumption rates increased with increasing oxygen concentrations up to about 40 μM and decreased slowly to the lowest rates at 140 μM oxygen. Filled circles represent rates obtained in type I chamber, while open circles represent rates obtained in type II chamber. (b) Oxygen consumption started immediately after the oxygenated medium was injected (final concentration 36 μM) into the culture. The highest rates were measured in the beginning of the monitoring period. (c) The effect of formate on the rate of oxygen consumption. The experiment was initiated by injection of oxygenation medium (final concentration 25 μM of oxygen). The immediate consumption rate of oxygen was 15 nmol O2 min-1 mg protein-1. After addition of formate (final concentration 20 mM) the oxygen consumption rate increased to 22 nmol O2 min-1 mg protein-1. [file 12864_2023_9297_MOESM8_ESM.pdf]

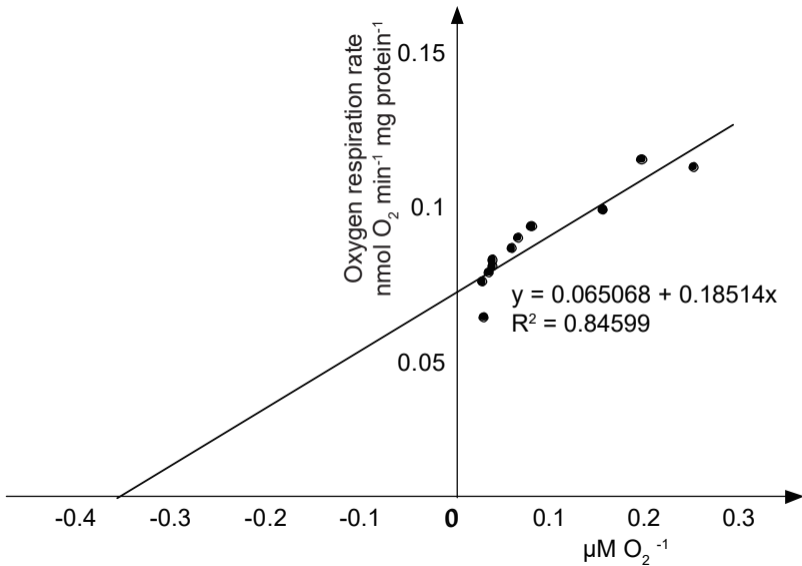

Supplement: Supplementary file 9 — Additional file 9: Figure S9. A Lineweaver-Burk plot constructed from the first eleven measurements shown in Fig. S8a. The X and Y intercepts are used to calculate Km and Vmax. [file 12864_2023_9297_MOESM9_ESM.pdf]

**a**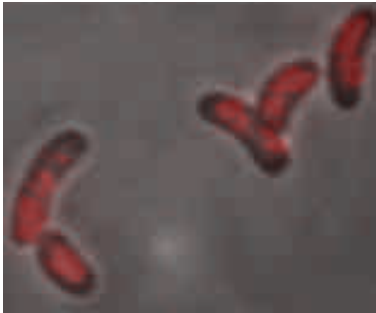**b**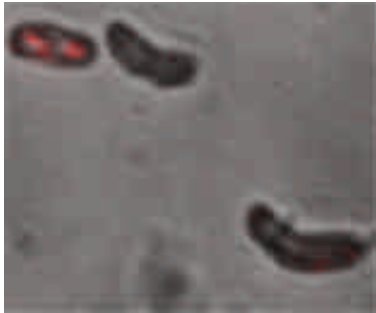

Supplement: Supplementary file 10 — Additional file 10: Figure S10. Cells of D. hansenii before (a) and after (b) exposure to 60 μM oxygen for 20 h. The cells were stained with Nile blue, which binds to polyhydroxyalkanoates. The red color represents areas in the cells, which were stained by Nile blue, indicating that the amount of polyhydroxyalcanoates decreased after exposure to oxygen. [file 12864_2023_9297_MOESM10_ESM.pdf]
