# Supplementary material for: Genomic insight of sulfate reducing bacterial genus Desulfofaba reveals their metabolic versatility in biogeochemical cycling
Source: BMC Genomics. 2023 Apr 19;24:209. doi: 10.1186/s12864-023-09297-2 (PMC10116758; doi:10.1186/s12864-023-09297-2)
Supplement: Supplementary file 6 — Additional file 6: Figure S6. Maximum likelihood phylogenetic tree of FeFe hydrogenases. Bootstrap values ≥ 75 are shown in circles. [file 12864_2023_9297_MOESM6_ESM.pdf]

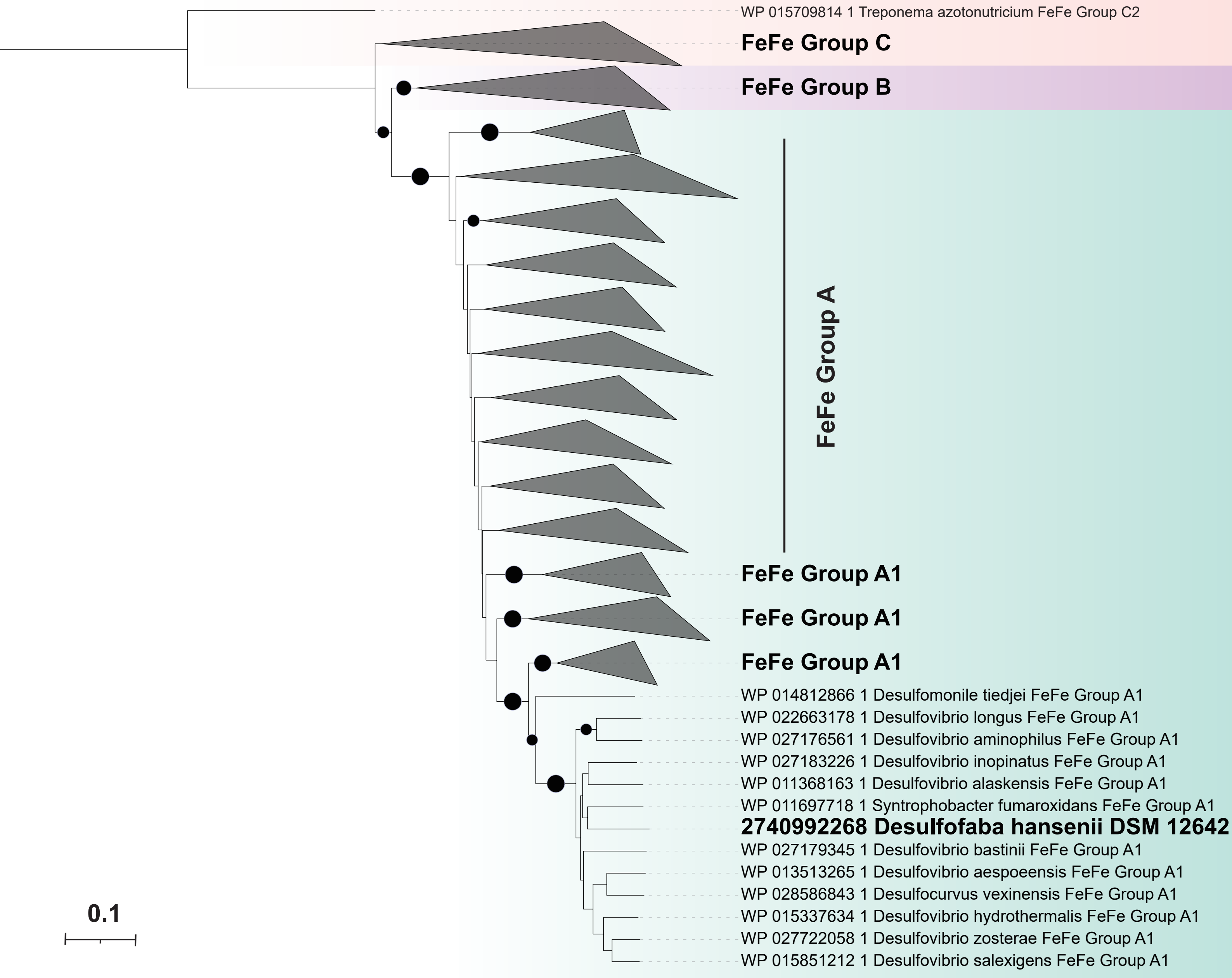
WP 015709814 1 Treponema azotonutricium FeFe Group C2
FeFe Group C
FeFe Group B
FeFe Group A
FeFe Group A1
FeFe Group A1
FeFe Group A1
WP 014812866 1 Desulfomonile tiedjei FeFe Group A1
WP 022663178 1 Desulfovibrio longus FeFe Group A1
WP 027176561 1 Desulfovibrio aminophilus FeFe Group A1
WP 027183226 1 Desulfovibrio inopinatus FeFe Group A1
WP 011368163 1 Desulfovibrio alaskensis FeFe Group A1
WP 011697718 1 Syntrophobacter fumaroxidans FeFe Group A1
2740992268 Desulfofaba hansenii DSM 12642
WP 027179345 1 Desulfovibrio bastinii FeFe Group A1
WP 013513265 1 Desulfovibrio aespoeensis FeFe Group A1
WP 028586843 1 Desulfocurvus vexinensis FeFe Group A1
WP 015337634 1 Desulfovibrio hydrothermalis FeFe Group A1
WP 027722058 1 Desulfovibrio zosterae FeFe Group A1
WP 015851212 1 Desulfovibrio salexigens FeFe Group A1
0.1
