# Supplementary material for: Genomic insight of sulfate reducing bacterial genus Desulfofaba reveals their metabolic versatility in biogeochemical cycling
Source: BMC Genomics. 2023 Apr 19;24:209. doi: 10.1186/s12864-023-09297-2 (PMC10116758; doi:10.1186/s12864-023-09297-2)
Supplement: Supplementary file 12 — Additional file 12. [file 12864_2023_9297_MOESM12_ESM.docx]

Supplementary Information for

**Genomic insight of sulfate reducing bacterial genus *Desulfofaba* reveals their metabolic versatility in biogeochemical cycling**

Ping Gao^1,2^, Xiaoting Zhang^3^, Xiaomei Huang^3^, Zhiyi Chen^3^, Angeliki Marietou^4^, Lars Holmkvist^4^, Lingyun Qu^1,2^, Kai Finster^4,5^, Xianzhe Gong^3^*

^1^ Key Laboratory of Marine Eco-Environmental Science and Technology, First Institute of Oceanography, Ministry of Natural Resources, Qingdao, 266061, PR China

^2^ Laboratory for Marine Fisheries Science and Food Production Processes, Pilot National Laboratory for Marine Science and Technology (Qingdao), Qingdao, 266237, PR China

^3^ Institute of Marine Science and Technology, Shandong University, Qingdao, 266237, PR China

^4^

Section for Microbiology, Department of Bioscience, Aarhus University, Aarhus 8000, Denmark

^5^ Stellar Astrophysics Center, Department of Physics and Astronomy, Aarhus University, Aarhus 8000, Denmark

Running title: Genome of a genus of microaerophilic sulfate reducer.

*Corresponding author: xianzhe.gong@gmail.com

This file includes:

Supplementary Materials and Methods

Supplementary Results and Discussion

Supplementary Figures S1-S10

Other Supplementary Information for this manuscript includes the following:

Description of Supplementary Dataset

Supplementary References

**Supplementary Materials and Methods**

*Preparation for Desulfofaba hansenii Culture*

The cultivation procedure for *Desulfofaba hansenii* strain P1 was described by Finster et al. [[1]](https://paperpile.com/c/o4dsZF/852l). For respiration experiments, 50 ml of a densely grown culture in the late exponential phase was transferred to a sterile 50-ml centrifuge tube with a removable rubber stopper in the lid and centrifuged at 5000 rpm for 15 min. The supernatant was removed, and the headspace of the centrifuge tube was flushed with N_2_. The cell pellet was resuspended in fresh sulfide- and oxygen-free medium and washed to remove residual sulfide. The washing medium was prepared under N_2_ and did not contain a reducing agent. Immediately after washing, the culture pellet was transferred to a gas-tight reaction chamber in which the oxygen respiration experiments were carried out. The presence of residual sulfide after the cell pellet had been washed was tested according to a Diamine method [[2]](https://paperpile.com/c/o4dsZF/1Ddt). The volume of 0.1 ml of the cell pellet was diluted in 0.5 ml 20% Zn-acetate and 9.4 ml of water. A volume of 0.5 ml of this solution was further diluted in 0.1 ml diamine solution and 1.0 ml of water. The solution was incubated in darkness for 30 min and absorbance was measured on a spectrophotometer at wavelength 670 nm.

*Reaction chambers*

Two reaction chambers for the study of oxygen consumption were used. Type I was operated with a fixed volume (Fig. S7a) consisting of an 8-ml glass vial sealed with a black rubber stopper. A Clark-type macro-electrode was inserted through an opening in the rubber stopper [[3]](https://paperpile.com/c/o4dsZF/W22n). The vial was inoculated with a syringe, and overflow and bubbles were collected with a second syringe. Bubbles were avoided in all incubations. Oxygen was introduced by the injection of oxygen saturated sterile medium, and the liquids were mixed with a magnetic stir-bar placed on the bottom of the vial. Due to its limited size, the type I chamber could only be used for rate measurements at oxygen concentrations of up to 40 μM. Tests at higher concentrations were carried out in a second chamber (Type II, Fig. S7b), in which the volume was regulated by a rubber piston. The piston was equipped with an inlet and an outlet pipe through which medium and substrates could be added or withdrawn (Fig. S7). To prevent inflow of oxygen into the chamber through the pipes, the space above the rubber piston was flushed with N_2_. The macro-electrode was inserted through a rubber stopper in the wall of the chamber. Mixing was achieved as in the Type I chamber. The macro-electrode was connected to a pico-ampere meter from which readings were transmitted to a plotter. Oxygen uptake rates were calculated from the slope of the plotter readings.

*Experimental procedure*

The washed pellet was suspended in 10 ml of anoxic propionate-containing medium. Prior to injection, the chambers were flushed with nitrogen gas. The medium was then injected to the chambers to collect data with stirrer on. The experiments were initiated by injection of variable amounts of air-saturated medium, after which the change in oxygen concentration was monitored. The oxygen consumption rate was measured at start concentrations ranging from 5 to 140 μM. In the beginning and in the end of the experiments, samples were taken for microscopic inspection of the cells. At the end of the experiment, the medium was collected, and the bacterial biomass was determined as protein, according to a modified Benedict-procedure [[4]](https://paperpile.com/c/o4dsZF/gxvk). Two ml of the culture was centrifuged at 5000 rpm for 15 min. The pellet was suspended in 2 ml of a 6% NaOH solution and incubated in a water bath at 80°C for 15 min to obtain cell lyses. The suspension was then centrifuged to remove inorganic precipitates. One ml of the supernatant was transferred to an Eppendorf tube and 50 μl of a Cu^2+^ solution was added. The Cu^2+^ solution was prepared as follows: 173 g sodium citrate and 100 g sodium carbonate was dissolved in 500 ml of water, and 17.3 g CuSO_4_⋅5H_2_O was dissolved in 100 ml of water. The two solutions were mixed, and water was added to a final volume of 1000 ml. The intensity of the formed complex was determined on a spectrophotometer at wavelength 330 nm.

In addition to varying oxygen concentrations, the influence of different organic substrates was tested. The substrate was injected after the culture had received oxygen-saturated medium. The initial oxygen concentration was between 21 and 34 μM and the substrate concentration was 20 mM. The following substrates were tested: fumarate, formate, ethanol, pyruvate, acetate, and butyrate. Acetate and butyrate did not serve as the substrates when *Desulfofaba* *hansenii* was grown as a sulfate reducer. Cultures without additional substrates served as controls.

*Test of the usage of internal storage compounds under oxic condition*

Cells were stained with Nile blue (1%) in order to elucidate the internal storage compounds. Cells were spread, dried in air, fixed on a slide and dyed in Nile blue for 20 min at 55 ºC. After staining, the slide was rinsed in acetic acid (8%) and water to remove additional dye. The slide was visualized with immersion oil on an epifluorescence microscope.

One hundred ml of the culture was centrifuged, washed, and diluted in 25 ml of anoxic propionate-containing medium. The cells were injected into a 100-ml glass bottle, flushed with N_2,_ and placed on a magnetic stirrer. Samples were taken during the experiment and stained with Nile blue. The first sample was taken before any oxygen was added and served as a control. Oxygen was added to the headspace, giving a concentration of approximately 60 μM in the media, and two samples were taken after 5 and 20 hours. The oxygen concentration in the glass bottle was kept constant during the experiment. Oxygen was measured in the headspace by injecting headspace-gas (300μL) into a gas chromatograph (ML GC 82-22 with TCD).

**Supplementary Results and Discussion**

*Desulfofaba* *hansenii* was able to reduce oxygen at all tested start concentrations (4-140 μM) (Fig. S8a). An upper limit for oxygen respiration was not determined. The lowest start concentration tested was 4 μM. At this start concentration, the rate of oxygen consumption was about 9 nmol O_2_ min^-1^ mg protein^-1^. The highest rate of 23 nmol O_2_ min^-1^ mg protein^-1^ was found at an oxygen start concentration of 36 μM. Above 60 μM, the oxygen consumption rate decreased with increasing oxygen concentrations (Fig. S8a). The lowest rate of about 5 nmol O_2_ min^-1^ mg protein^-1^ was determined at 140 μM. At all concentrations, oxygen consumption started immediately after the culture was supplemented with oxygen-containing medium (Fig. S8b). The highest rates were typically recorded immediately after oxygen addition. Repeated injections of oxygen in the lower concentration range (up to 30 μM) only slightly affected the oxygen consumption rates determined immediately after oxygen addition. Repeated exposure to intermediate oxygen concentrations (140 μM) affected the respiration rates in a negative way, indicating that the organism is stressed by the presence of oxygen.

With respiration rates ranging from 5 to 22.8 nmol O_2_ min^-1^ mg protein^-1^, *Desulfofaba* *hansenii* consumes oxygen at rates that are situated at the low end of reported rates [[5–8]](https://paperpile.com/c/o4dsZF/fci2+NjiG+AinN+id5H). Kuhnigk et al. [[8]](https://paperpile.com/c/o4dsZF/id5H) reported respiration rates as high 1570 nmol O_2_ min^-1^ mg total protein^-1^ in *Desulfovibrio*, a rate that is higher than rates reported for most aerobic bacteria. In contrast to the sulfate reducers investigated by Cypionka and co-workers [[9]](https://paperpile.com/c/o4dsZF/VMYt), aerobic respiration rates of *Desulfofaba* *hansenii* increased with increasing oxygen concentrations. The highest respiration rate was measured at an oxygen concentration of about 40 μM. It has to be taken into consideration that the residual amount of sulfide after washing (8.8 -10 μM) is able to react with oxygen chemically thereby decreasing the oxygen concentration. The competition between a chemical and a biological removal of oxygen is, however, only critical at low oxygen concentrations and, in particular, during the first run of repeated oxygen injections. The most likely product of the chemical reaction is elemental sulfur. The highest amount of measured residual sulfide was 10μM, which can remove about 5 μM of O_2_. The complete and repeated oxygen consumption at higher oxygen concentrations can only be explained by the active oxygen consumption by *Desulfofaba* *hansenii*. Our data indicate that aerobic respiration by *Desulfofaba* *hansenii* can be described by a Michealis-Menten type of kinetics, and, hence, we determined the apparent *K_m_* and *V_max_* of oxygen respiration from a Lineweaver-Burk plot (Fig. S9). The apparent *K_m_* value for oxygen was 2.84 μM and *V_max_* was 15.36 nmol O_2_ min^-1^ mg protein^-1^. Previously reported apparent *K_m_* values for oxygen uptake by aerobic bacteria were within a wide range (5.2-135.3 μM) and higher [[10]](https://paperpile.com/c/o4dsZF/cl7G) than the value determined in this study. Different types of bacteria have different ranges of apparent *K_m_* values e.g., reported apparent *K_m_* values in the ammonium oxidizer *Nitrosomonas europaea* range from 5-15 μM, and in the nitrite-oxidizing bacterium *Nitrobacter winogradskyi* range from 22-166 μM [[11]](https://paperpile.com/c/o4dsZF/skwc). Lower apparent *K_m_* values are found in heterotrophic bacteria; 0.018-0.2 μM in *Escherichia coli* [[12]](https://paperpile.com/c/o4dsZF/tA1n), 1.4-5.6 μM in *Pseudomonas chlororaphis* [[13]](https://paperpile.com/c/o4dsZF/htUw), and below 0.01 µM in marine bacteria *Roseobacter denitrificans*, *Idiomarina loihiensis*, and *Marinobacter daepoensis* [[14]](https://paperpile.com/c/o4dsZF/SkvJ).

Formate stimulated oxygen consumption (22 nmol O_2_ min^-1^ mg protein^-1^) by about 50% compared to the control (15 nmol O_2_ min^-1^ mg protein^-1^) (Fig. 8c). This is inconsistent with the finding that, in the microaerophilic bacterium *Campylobacter jejuni*, oxidation of formate had a negative effect on oxygen tolerance [[15]](https://paperpile.com/c/o4dsZF/CA05), as the oxidation of formate generated H_2_O_2_.  Propionate, which was routinely used when the organism was grown with sulfate as electron acceptor, did not stimulate oxygen respiration, nor did fumarate or ethanol. When no substrate was added, *Desulfofaba* *hansenii* was able to reduce oxygen at initial concentrations between 25 and 34 μM, at respiration rates between 14 and 17 nmol O_2_ min^-1^ mg protein^-1^. Generally, the measured rates of oxygen respiration were in the same range whether substrates were present or not. This indicates that *Desulfofaba* *hansenii* uses a storage compound, which is mobilized during aerobic respiration. Electron micrographs have clearly shown that *Desulfofaba* *hansenii* is tightly packed with spherical inclusions, which may serve this purpose [[1]](https://paperpile.com/c/o4dsZF/852l).

Staining *Desulfofaba* *hansenii* with Nile blue demonstrated numerous spherical inclusions (Fig. S10). After 5 hours of exposure to oxygen (60 μM) a slight decrease in the brightness and number of granules were visually estimated. After 20 hours, the general picture was a decrease in brightness, size and number of the granules, and some cells had lost the granules (Fig. S10). It suggests that the inclusions contain polyhydroxyalkanoates. In recent studies it has been demonstrated that polyglucose was involved in aerobic respiration in some *Desulfovibrio* strains [[16–18]](https://paperpile.com/c/o4dsZF/NTFe+Hd4Z+1eSH). Oxygen respiration only took place when polyglucose was present in the cell [[15]](https://paperpile.com/c/o4dsZF/CA05). The actual mechanism by which polyglucose is degraded is still not clarified, but studies indicate that aerobic conversion is mediated by the glycolytic pathway [[15]](https://paperpile.com/c/o4dsZF/CA05). In the case of *Desulfofaba* *hansenii*, polyhydroxyalkanoate and not polyglucose may support aerobic respiration, which our results with Nile blue staining indicate (Fig. S10). It has been shown that bacteria utilize polyhydroxyalkanoate for growth under aerobic conditions in an activated sludge system [[19]](https://paperpile.com/c/o4dsZF/nyCt). Our report on the utilization of polyhydroxyalkanoate in *Desulfofaba* *hansenii* under aerobic conditions would be the first example in a sulfate reducer. However, future physiological experiments concerning the degradation of these compounds during respiration with oxygen and characterization of pathways are still needed in order to evaluate their role.

**References:**

[1. Finster K, Thomsen TR, Ramsing NB. Desulfomusa hansenii gen. nov., sp. nov., a novel marine propionate-degrading, sulfate-reducing bacterium isolated from Zostera marina roots. Int J Syst Evol Microbiol. 2001;51 Pt 6:2055–61.](http://paperpile.com/b/o4dsZF/852l)

[2. Cline JD. Spectrophotometric determination of hydrogen sulfide in natural waters1. Limnol Oceanogr. 1969;14:454–8.](http://paperpile.com/b/o4dsZF/1Ddt)

[3. Revsbech NP. An oxygen microsensor with a guard cathode. Limnol Oceanogr. 1989;34:474–8.](http://paperpile.com/b/o4dsZF/W22n)

[4. Brewer, / Pesce JM, / Ashworth AJ, R.B. Experimentelle Methoden in der Biochemie. Munich, Germany: Urban & Fischer in Elsevier; 1977.](http://paperpile.com/b/o4dsZF/gxvk)

[5. Krekeler D, Sigalevich P, Teske A, Cypionka H, Cohen Y. A sulfate-reducing bacterium from the oxic layer of a microbial mat from Solar Lake (Sinai), Desulfovibrio oxyclinae sp. nov. Arch Microbiol. 1997;167:369–75.](http://paperpile.com/b/o4dsZF/fci2)

[6. Dannenberg S, Kroder M, Dilling W, Cypionka H. Oxidation of H2, organic compounds and inorganic sulfur compounds coupled to reduction of O2 or nitrate by sulfate-reducing bacteria. Arch Microbiol. 1992;158:93–9.](http://paperpile.com/b/o4dsZF/NjiG)

[7. Krekeler D, Teske A, Cypionka H. Strategies of sulfate-reducing bacteria to escape oxygen stress in a cyanobacterial mat. FEMS Microbiol Ecol. 1998;25:89–96.](http://paperpile.com/b/o4dsZF/AinN)

[8. Kuhnigk T, Branke J, Krekeler D, Cypionka H, König H. A Feasible Role of Sulfate-Reducing Bacteria in the Termite Gut. Syst Appl Microbiol. 1996;19:139–49.](http://paperpile.com/b/o4dsZF/id5H)

[9. Cypionka H, Widdel F, Pfennig N. Survival of sulfate-reducing bacteria after oxygen stress, and growth in sulfate-free oxygen-sulfide gradients. FEMS Microbiol Ecol. 1985;1:39–45.](http://paperpile.com/b/o4dsZF/VMYt)

[10. Laanbroek HJ, Bodelier PLE, Gerards S. Oxygen consumption kinetics of Nitrosomonas europaea and Nitrobacter hamburgensis grown in mixed continuous cultures at different oxygen concentrations. Arch Microbiol. 1994;161:156–62.](http://paperpile.com/b/o4dsZF/cl7G)

[11. Laanbroek HJ, Gerards S. Competition for limiting amounts of oxygen between Nitrosomonas europaea and Nitrobacter winogradskyi grown in mixed continuous cultures. Arch Microbiol. 1993;159:453–9.](http://paperpile.com/b/o4dsZF/skwc)

[12. Rice CW, Hempfling WP. Oxygen-limited continuous culture and respiratory energy conservation in Escherichia coli. J Bacteriol. 1978;134:115–24.](http://paperpile.com/b/o4dsZF/tA1n)

[13. Bodelier PLE, Laanbroek HJ. Oxygen uptake kinetics of Pseudomonas chlororaphis grown in glucose- or glutamate-limited continuous cultures. Arch Microbiol. 1997;167:392–5.](http://paperpile.com/b/o4dsZF/htUw)

[14. Gong X, Garcia-Robledo E, Schramm A, Revsbech NP. Respiratory Kinetics of Marine Bacteria Exposed to Decreasing Oxygen Concentrations. Appl Environ Microbiol. 2015;82:1412–22.](http://paperpile.com/b/o4dsZF/SkvJ)

[15. Hoffman PS, George HA, Krieg NR, Smibert RM. Studies of the microaerophilic nature of Campylobacter fetus subsp. jejuni. II. Role of exogenous superoxide anions and hydrogen peroxide. Can J Microbiol. 1979;25:8–16.](http://paperpile.com/b/o4dsZF/CA05)

[16. Fareleira P, Legall J, Xavier AV, Santos H. Pathways for utilization of carbon reserves in Desulfovibrio gigas under fermentative and respiratory conditions. J Bacteriol. 1997;179:3972–80.](http://paperpile.com/b/o4dsZF/NTFe)

[17. van Niel EW, Gottschal JC. Oxygen Consumption by Desulfovibrio Strains with and without Polyglucose. Appl Environ Microbiol. 1998;64:1034–9.](http://paperpile.com/b/o4dsZF/Hd4Z)

[18. van Niel EWJ, Pedro Gomes TM, Willems A, Collins MD, Prins RA, Gottschal JC. The role of polyglucose in oxygen-dependent respiration by a new strain of Desulfovibrio salexigens. FEMS Microbiol Ecol. 1996;21:243–53.](http://paperpile.com/b/o4dsZF/1eSH)

[19. Karahan O, Orhon D, van Loosdrecht MCM. Simultaneous storage and utilization of polyhydroxyalkanoates and glycogen under aerobic conditions. Water Sci Technol. 2008;58:945–51.](http://paperpile.com/b/o4dsZF/nyCt)

**Figure S1.** A maximum likelihood phylogenetic tree of xx genomes including the 3 *Desulfofaba* genomes. The phylogeny is based on 120 concatenated ribosomal protein encoding genes identified using GTDB-tk. Acidobacteria were set as the outgroup.

**Figure S2.** Maximum likelihood phylogenetic tree of 16S rRNA gene.

**Figure S3.** Hierarchical clustering heatmap using pheatmap package in R based on average amino acids identity (AAI) for each genome pair.

**Figure S4.** Carbohydrate-active enzymes (CAZyme) and peptidase encoded by *Desulfofaba* genus. (a) CAZymes include carbohydrate esterase (CE), glycoside hydrolase (GH), and polysaccharide lyase (PL). (b) Peptidases are classified by family as aspartic (A), cysteine (C), unassigned inhibitors (I), metallo (M), asparagine (N), serine (S), threonine (T), and unknown (U) by the MEROPS database. Sizes of the circle denote the number of gene copies in the genome. The number on top of the circle represents the number of sequences identified with potential secretion signal using PSORTb v3.0.

**Figure S5.** Maximum likelihood phylogenetic tree of NiFe hydrogenases. Bootstrap values ≥ 75 are shown in circles.

**Figure S6.** Maximum likelihood phylogenetic tree of FeFe hydrogenases. Bootstrap values ≥ 75 are shown in circles.

**Figure S7.** Schematic drawing of the reaction chambers used in this study. Type I chamber (a) was used at oxygen concentrations between 0 and 36 μM; type II chamber (b) was used up to 140 μM.

**Figure S8.** Oxygen respiration under different conditions. (a) Oxygen consumption rates under different initial oxygen concentrations. The oxygen consumption rates increased with increasing oxygen concentrations up to about 40 μM and decreased slowly to the lowest rates at 140 μM oxygen. Filled circles represent rates obtained in type I chamber, while open circles represent rates obtained in type II chamber. (b) Oxygen consumption started immediately after the oxygenated medium was injected (final concentration 36 μM) into the culture. The highest rates were measured in the beginning of the monitoring period. (c) The effect of formate on the rate of oxygen consumption. The experiment was initiated by injection of oxygenation medium (final concentration 25 μM of oxygen). The immediate consumption rate of oxygen was 15 nmol O_2_ min^-1^ mg protein^-1^. After addition of formate (final concentration 20 mM) the oxygen consumption rate increased to 22 nmol O_2_ min^-1^ mg protein^-1^.

**Figure S9.** A Lineweaver-Burk plot constructed from the first eleven measurements shown in Figure S8a. The X and Y intercepts are used to calculate *K_m_* and *V_max_*.

**Figure S10.** Cells of *D*. *hansenii* before (a) and after (b) exposure to 60 μM oxygen for 20 h. The cells were stained with Nile blue, which binds to polyhydroxyalkanoates. The red color represents areas in the cells, which were stained by Nile blue, indicating that the amount of polyhydroxyalcanoates decreased after exposure to oxygen.

**Description of Supplementary Dataset**

Detailed annotation of the three *Desulfofaba* genomes.
